# GALNT6 promotes invasion and metastasis of human lung adenocarcinoma cells through O-glycosylating chaperone protein GRP78

**DOI:** 10.1038/s41419-020-2537-6

**Published:** 2020-05-11

**Authors:** Jing Song, Wenwen Liu, Jianzhen Wang, Junxia Hao, Yingyan Wang, Xin You, Xiaohui Du, Yang Zhou, Jing Ben, Xinri Zhang, Mingliang Ye, Qi Wang

**Affiliations:** 1 Department of Respiratory Medicine, The Second Hospital, Dalian Medical University, No. 467 Zhongshan Road, Dalian, Liaoning 116023 China; 2 Department of Respiratory and Critical Care Medicine, The First Hospital, Shanxi Medical University, No. 85Jiefang South Road, Taiyuan, Shanxi 030001 China; 3 Laboratory Center for Diagnostics, Dalian Medical University, No. 9 West Section Lvshun South Road, Dalian, Liaoning 116044 China; 4 CAS Key Laboratory of Separation Science for Analytical Chemistry, Dalian Institute of Chemical Physics, Chinese Academy of Science, No. 457 Zhongshan Road, Dalian, Liaoning 116023 China; 5 Department of Scientific Research Center, The Second Hospital, Dalian Medical University, No. 467 Zhongshan Road, Dalian, Liaoning 116023 China

**Keywords:** Non-small-cell lung cancer, Metastasis

## Abstract

Lung adenocarcinoma remains a threat to human health due to its high rate of recurrence and distant metastasis. However, the molecular mechanism underlying lung adenocarcinoma metastasis remains yet incompletely understood. Here, we show that upregulated expression of polypeptide *N*-acetylgalactosaminyltransferase6 (GALNT6) in lung adenocarcinoma is associated with lymph node metastasis and poor prognosis. In lung adenocarcinoma cells, GALNT6 over-expression promoted epithelial–mesenchymal transition (EMT), wound healing, and invasion which could be significantly reversed by GALNT6 silencing. GALNT6 silencing also mitigated the metastasis of lung adenocarcinoma and prolonged the survival of xenograft tumor-bearing mice. Furthermore, GALNT6 directly interacted with, and O-glycosylated chaperone protein GRP78, which promoted EMT by enhancing the MEK1/2/ERK1/2 signaling in lung cancer cells. Therefore, GALNT6 is emerging as novel positive regulator for the malignancy of human lung adenocarcinoma. Targeting GALNT6-GRP78-MEK1/2/ERK1/2 may thus represent a new avenue to develop therapeutics against lung cancer metastasis.

## Introduction

Lung adenocarcinoma is a common malignancy in the world, and has a high mortality rate^1^. Currently, although standard treatments for lung adenocarcinoma have achieved some progress, its high rates of recurrence and distant metastasis make it a major threat to patient health^2–6^. Unfortunately, the molecular mechanisms underlying lung adenocarcinoma metastasis have not been well clarified. Therefore, it is important to better understand the metastatic process of lung adenocarcinoma and uncover more therapeutic targets.

Polypeptide *N*-acetylgalactosaminyltransferase 6 (GALNT6) is an enzyme for O-glycosylation and its expression is increased in some human cancers, including lung adenocarcinoma^7–10^. Previous studies suggest that GALNT6 may be important for cancer formation, progression, metastasis, and prognosis^10–14^. GALNT6 expression is highly upregulated in colon adenocarcinoma but absent in normal-appearing adjacent colon tissue, suggesting that abnormal expression of GALNT6 may be an early event in colon carcinogenesis^8^. High GALNT6 expression is associated with the recurrence, lymph node metastasis, and chemoresistance of ovarian cancer^11^. Furthermore, GALNT6 is a potential biomarker for breast cancer progression and metastasis^12,13^, and an independent prognostic factor for the poor survival of gastric cancer patients^14^. In addition, GALNT6 is implicated in the epithelial–mesenchymal transition (EMT) process which is crucial for cancer metastasis^15^. Currently, it is unclear how GALNT6 regulates the EMT process to promote invasion and metastasis of lung adenocarcinoma.

Glucose-regulated protein 78 (GRP78) is a molecular chaperone mainly expressed in endoplasmic reticulum and promotes the folding and oligomerization of proteins. GRP78 is increased in some cancers and is associated with tumor cell survival, chemoresistance, and poor patient prognosis^16–20^. Furthermore, GRP78 promotes the invasion and metastasis of gastric, prostate, and breast cancers^21–23^. A recent study reveals that GALNT6 O-glycosylates and stabilizes GRP78 in breast cancer cells, which may be critical for its subcellular localization and anti-apoptotic function^24^. However, it is unclear whether GALNT6 also O-glycosylates and stabilizes GRP78 in lung adenocarcinoma cells and to regulate EMT and metastasis.

In this study, we therefore characterized GALNT6 expression in human lung adenocarcinoma tissues, examined the impact of GALNT6 expression on EMT and metastasis of cancer in cellular and animal experiments, and explored whether the molecular mechanism maybe through O-glycosylating and stabilizing GRP78. Our results may contribute to the development of potential strategies to target GALNT6/GRP78 and suppress metastasis of lung adenocarcinoma.

## Materials and methods

### Human tissue microarray

Lung adenocarcinoma tissue microarrays (HLugA180Su05,Outdo Biotech, Shanghai, China) contained 90 pairs of surgical lung adenocarcinoma and adjacent non-tumor specimens(median age: 61.5 years; range 30–84 years; 43 females) in two tissue array blocks and were collected between July 2004 and June 2009. All cases were diagnosed and staged by the 7th edition International Union Against Cancer/American Joint Committee on Cancer TNM classification. Those patients (except for five cases with incomplete records) were followed-up between 1 and 121 months post-surgery with a median following-up period of 39 months. The experimental protocol was approved by the Ethics Review Committee of the Second Hospital of Dalian Medical University, and this study was conducted in compliance with ethical and safe research practices involving human subjects or tissues. Informed consent was obtained from all subjects.

### Immunohistochemistry (IHC)

GALNT6 expression in each tissue was examined by IHC. Briefly, tissue sections (3 µm) were deparaffinized, rehydrated, incubated with 3% H_2_O_2_ in methanol and subjected to antigen retrieval by EDTA buffer. The sections were blocked with 5% bovine serum albumin (BSA), probed with anti-GALNT6 (1:100, ab151329, Abcam), anti-GRP78 (1:100, ab21685, Abcam), or anti-phospho-ERK1/2 (1:100, #4370, CST) at 4 °C overnight. The sections were reacted with biotinylated secondary antibodies, and detected using the Streptavidin-Peroxidase IHC assay kit and DAB (ZSGB-bio, China). Immunostaining was evaluated by two certified pathologists in a blinded manner and scored, according to the staining intensity (negative staining: 0 point; weak staining: 1 point; moderate staining: 2 point; and strong staining: 3 point) multiplied by the percentage of stained cells (positive cells ≤ 25% of the cells: 1 point; 26–50% of the cells: 2 point; 51–75% of the cells: 3 point; ≥75% of the cells: 4 point). The median value of GALNT6 scores was employed to determine the cutoff. Tumors with GALNT6 scores lower or equal to the median were designated as“low expression”, whereas those with scores higher than the median were designated as “high expression”. The positive control of GALNT6 expression was defined according to lung cancer from the website (http://www.proteinatlas.org).

### Analysis of Kaplan–Meier Plotter database

Open the web page of the Kaplan–Meier Plotter (KM Plotter) database (http://www.kmplot.com), select mRNA lung cancer of all the cancer type database and enter the gene symbol GALNT6. Then set the data analysis parameters, lung adenocarcinoma was selected for the histological type of lung cancer, and the median was selected for the cutoff value. Tumors with GALNT6 mRNA expression lower or equal to the median were designated as “low expression”, whereas those with mRNA expression higher than the median were designated as “high expression”. Finally, click on the “draw Kaplan–Meier plot” to generate the result of Kaplan–Meier analysis in the database.

### Cell culture

Human lung adenocarcinoma A549, H1299, SPCA-1, PC9, NCI-H1975, NCI-H522, and BEAS-2B cells were purchased from the Chinese Academy of Medical Sciences (Beijing, China) and were cultured in RPMI1640 medium containing 10% fetal bovine serum (FBS) at 37 °C in5% CO_2_. These cells were authenticated by STR Genotyping at Shanghai Biowing Applied Biotechnology.

### Plasmids, siRNA, and transfection

A549 and H1299 cells were transduced with lentivirus of LV6-GALNT6, LV6-GPR78 or control LV6-NC (GenePharma, Shanghai, China) and treated with polybrene to induce stable GALNT6 and GRP78 over-expression. The target sequences of GALNT6 and GRP78 were referred to its genetic sequence in the PubMed (https://www.ncbi.nlm.nih.gov/gene). SPCA-1 and PC9 cells were transduced with the lentivirus of LV16-GALNT6 (expressing specific shRNA) or its control LV16-NC (GenePharma) to silence GALNT6. The target sequences of GALNT6 shRNA1 and shRNA2 were 5′-GCACTGTTTCAATGCCTTTGC-3′, and 5′-GGATGGACAGCTACAAGAAGA-3′, respectively. SPCA-1 and PC9 cells were transfected with GRP78-specific siRNA (1: 5′-UCUUUCCCAAAUAAGCCUCTT-3′ or siRNA-2: 5′-AGUUUGGUCAUGACACCUCTT-3′) or control scrambled siRNA (GenePharma) using lipofectamine 2000 for 48 h.

### Confocal immunofluorescence

A549, H1299, SPCA-1, and PC9 cells were cultured on the Glass Bottom Cell Culture Dish for 24 h and then were fixed with 4% paraformaldehyde, permeabilized with 0.25% of Triton X-100 (Solarbio), and treated with 5% BSA. After being washed, the cells were probed with 1:100 diluted antibodies against GALNT6 (sc-100755, Santa Cruz Biotechnology), E-cadherin (13-1700, Invitrogen), N-cadherin (sc-393933, Santa Cruz Biotechnology), Slug (ab27568, Abcam), GRP78 (ab21685, Abcam) overnight at 4 °C. Subsequently, the cells were incubated with fluorophore-conjugated secondary antibodies (1:100, Proteintech) for 1 h and stained with DAPI (Sigma) for nuclei, followed by photoimaging under a confocal laser scanning microscope (Leica DM14000B).

### Western blot

Cells in experimental and control groups were lyzed and centrifuged. After determined the protein concentrations, the cell lysate samples (30 μg/lane) were separated by sodium dodecyl sulfate polyacrylamide gel electrophoresis (SDS-PAGE) on 10% gels and transferred onto NC membranes. The membranes were blocked with 5% BSA in TBST and immunoblotted by the primary antibodies. The primary antibodies used in this study were:anti-GALNT6 (1:1000, sc-100755, Santa Cruz Biotechnology), anti-E-cadherin (1:500, 13-1700, Invitrogen), anti-N-cadherin (1:200, sc-393933, Santa Cruz Biotechnology), anti-Slug (1:500, ab27568, Abcam), anti-GRP78 (1:1000, ab21685, Abcam), anti-phospho-MEK1/2 (1:1000, #9154, CST), anti-MEK1/2 (1:1000, ab178876, Abcam), anti-phospho-ERK1/2 (1:1000, #4370, CST), anti-ERK1/2 (1:1000, #4695, CST), biotinylated anti-Viciavillosa agglutinin (specific to GalNAc-Ser/Thr) (VVA, 1:1000, B-1235, Vector) and anti-GAPDH (1:2000, 10494-1-AP, Proteintech). The secondary antibodies were HRP-conjugated Affinipure Goat Anti-Mouse IgG (1:5000, SA00001-1, Proteintech), HRP-conjugated Affinipure Goat Anti-Rabbit IgG (1:5000, SA00001-2, Proteintech) and HRP-streptavidin (1:10000, SA-5014, Vector). The protein bands were visualized by enhanced chemiluminescent reagents (Advansta) and the intensity of protein band was quantified by ImageJ software.

### Wound healing assay

A549, H1299, SPCA-1, and PC9 cells were cultured in 6-well plates. When the cells grew to 70–80% confluency, wounds were created using a sterile 200 μl pipette tip. The wound areas were photoimaged at 0 and 24 h post wounding.

### Invasion assay

Cells were seeded into Matrigel-coated top chamber of 24-well transwell plates (8-μm pore, #3422, Corning) (2.5 × 10^5^ cells/well) and cultured in serum-free medium. The bottom chambers contained complete medium and cultured for 24 h. The non-invading cells on the supper surface of the top chambers were removed using a cotton swab and the invaded cells onto the bottom surface of the top chambers were fixed in 4% paraformaldehyde and stained with 2% crystal violet, followed by photoimaged under a microscope (Leica, TCSSP5II). The invaded cells in five fields selected randomly in one image were counted in a blinded manner.

### Animal study

Animal study was approved by the Animal Research and Care Committee of Dalian Medical University. Female BALB/c nude mice (4–6 weeks inage) were obtained from Vital River Laboratory Animal Technology (Beijing) and housed in a specific pathogen-free facility with free access to autoclaved food and water. Each mouse was anesthetized using ketamine (100 mg/kg body weight; Sigma, USA) and xylazine (10 mg/kg body weight; Sigma, USA), and implanted with PC9/shNC or PC9/shG6-1 cells (10^6^ cells/mouse in saline) which were engineered to stably express Luciferase protein through transfection of a plasmid vector into its left ventricle. The metastasis of tumors in mice was monitored by bioluminescence imaging (BLI). Briefly, the mice were anesthetized and injected retro-orbitally with D-Luciferin (150 mg/kg body weight; Promega, USA) followed by imaging in an IVIS Spectrum Xenogen machine (Caliper Life Sciences). The Living Image software (version 2.50) was used to analyze the bioluminescence images to assess metastasis.

### Co-immunoprecipitation

A549 cells were lyzed and their cell extracts were reacted with antibodies against GALNT6 or GRP78 in rotating at 4 °C for 1 h and the immunocomplex was precipitated with Protein A columns (#635721, Capturem IP & Co-IP Kit, Takara Bio, USA). After being washed, the proteins were separated by SDS-PAGE, followed by Western blot analysis using anti-GALNT6 and anti-GRP78.

### qRT-PCR

Total RNA was extracted from different groups of cells using RNA prep Pure Cell Kit (DP430, Transgen Biotech, Beijing, China), according to manufacturer’s instructions and reversely transcribed into cDNA using Fast Quant RT Kit (KR106, Transgen Biotech, Beijing, China). The relative levels of human GRP78 mRNA transcripts to GAPDH were quantified in triplicate by qRT-PCR using Super Real PreMix Plus (FP205, Transgen Biotech, Beijing, China) and specific primers. The primer sequences were GRP78-F 5′-CATCACGCCGTCCTATGTCG-3′ and GRP78-R5’-CGTCAAAGACCGTGTTCTCG-3′; GAPDH-F 5′-CATGAGAAGTATGACAACAGCCT-3′ and GAPDH-R 5′-AGTCCTTCCACGATACCAAAGT-3′. All data were analyzed by 2^−ΔΔCt^.

### Statistical analysis

Data are expressed as the mean ± SD and analyzed by GraphPad Prism (version 5.0; GraphPad Software, San Diego, CA, USA). The data among different experimental groups were compared by analysis of variance (ANOVA), Student’s *t* test or Chi-square test where applicable. Survival curves were estimated by Kaplan–Meier method and analyzed by the log rank test. Statistical significance was defined when a *P*-value was <0.05.

## Results

### Increased GALNT6 expression is associated with poor prognosis of lung adenocarcinoma patients

**Fig. 1:**
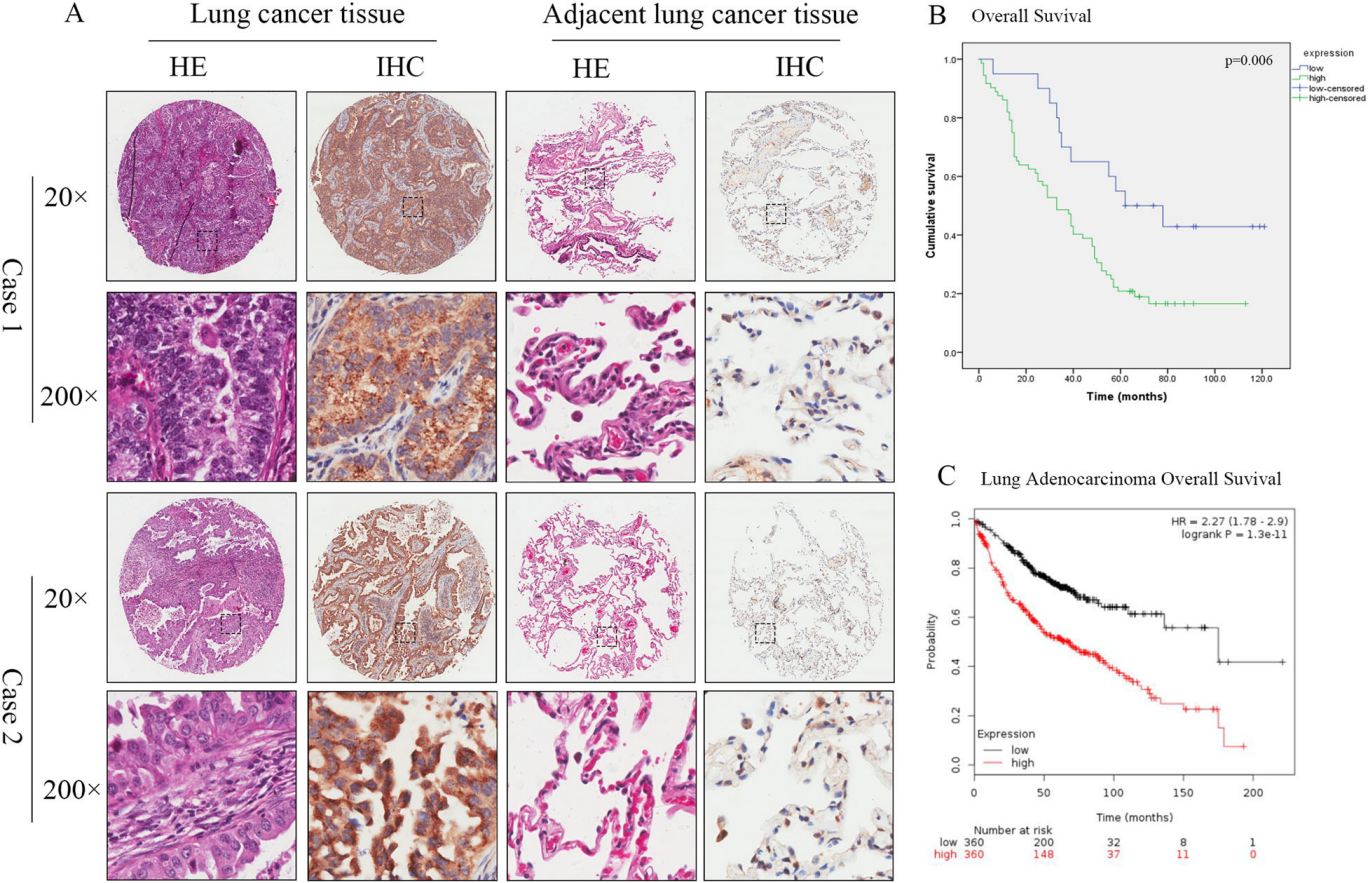
Increased GALNT6 expression is correlated with poor prognosis of lung adenocarcinoma patients. GALNT6 expression in 85 pairs of lung adenocarcinoma and their adjacent non-tumor tissues was examined by IHC. The overall survival of patients with high or low GALNT6 protein expressing lung adenocarcinoma was stratified and analyzed. In addition, the overall survival of 720 patients with high or low GALNT6 mRNA expressing lung adenocarcinoma in KM Plotter database was analyzed. Data are representative images or survival curves of patients. **a** IHC analysis of GALNT6 expression. **b** Kaplan–Meier analysis of the overall survival of 85 lung adenocarcinoma patients. **c** Kaplan–Meier analysis of the overall survival of 720 lung adenocarcinoma patients in KM Plotter database.

Numerous studies indicate that abnormal expression of GALNT6 may be important for cancer formation, progression, metastasis and prognosis^10–14^. To observe the role of GALNT6 in the development and progression of human lung adenocarcinoma, 85 pairs of lung adenocarcinoma and adjacent non-tumor tissues were examined for GALNT6 expression. GALNT6 was mainly located in the cytoplasm of tumor cells and 77.64% of lung adenocarcinoma tissues expressed high levels of GALNT6 as compared to 7% of adjacent non-tumor tissues (*P* < 0.001) (Fig. 1a and Table 1). Stratification analyses showed that higher GALNT6 expression in lung adenocarcinoma was significantly correlated with higher T stage (P = 0.017), positive lymph node metastasis (*P* = 0.024) and higher TNM stages (*P* = 0.001), but not with patient age, gender and differentiation grade (Table 2).

**Table 1:** Differential expression of GALNT6 in cancerous and lung tissues.

		GALNT6 expression			
	*n*	High	Low	Chi-square value	*p*-value
Lung adenocarcinoma	85	66	19	86.735	<0.001*
Lung tissues	85	6	79		

^*^Statically significance (*p* < 0.05).

**Table 2:** Correlation between GALNT6 expression and clinicopathological characteristics.

		GALNT6 expression				
	Variables	High	low	total	χ2	*p*-value
Age					0.638	0.425
	≤60	35	8	43		
	>60	38	13	51		
Sex					0.480	0.488
	Female	32	11	43		
	male	41	10	51		
Grade					1.766	0.184
	I/II	48	17	65		
	III	25	4	29		
T stage					5.682	0.017*
	T1/T2	51	20	71		
	T3/T4	22	1	23		
N stage					5.074	0.024*
	N0	25	14	39		
	N1/N2/N3	32	5	37		
	null			18		
M stage					0.486	0.486
	M0	72	20	92		
	M1	1	0	1		
	null			1		
TNM stage					13.814	<0.001*
	Ι/II	29	17	46		
	III/IV	41	2	43		
	null			5		

^*^Statically significance (*p* < 0.05).

Kaplan–Meier analyses revealed that patients with high GALNT6 expressing lung adenocarcinoma had a significantly shorter survival than those with low GALNT6 expression (*P* = 0.006, Fig. 1b). Similar findings were obtained in 720 lung adenocarcinoma patients (Fig. 1c, *P* = 1.3e−11) from in KM Plotter database. Hence, high GALNT6 expression level was associated with poor prognosis of human lung adenocarcinoma.

### GALNT6 promotes EMT of lung adenocarcinoma cells

It was previously reported that GALNT6 is implicated in the EMT process which is crucial for cancer metastasis and poor prognosis^15^. To examine the role of GALNT6 in the EMT progression of lung adenocarcinoma, firstly, we measured its expression in non-tumor bronchial epithelial BEAS-2B and different lung adenocarcinoma cells, the result showed that higher levels of GALNT6 proteins were in NCI-H1975, SPCA-1 and PC9 tumor cells, but low levels were in BEAS-2B, lung adenocarcinoma A549, NCI-H1299 and NCI-H522 cells (Supplementary Fig. 1A). We then used lentivirus technology to induce stable GALNT6 over-expression in A549 and H1299 cells and GALNT6 silencing in SPCA-1 and PC9 cells (Supplementary Fig. 1B).

**Fig. 2:**
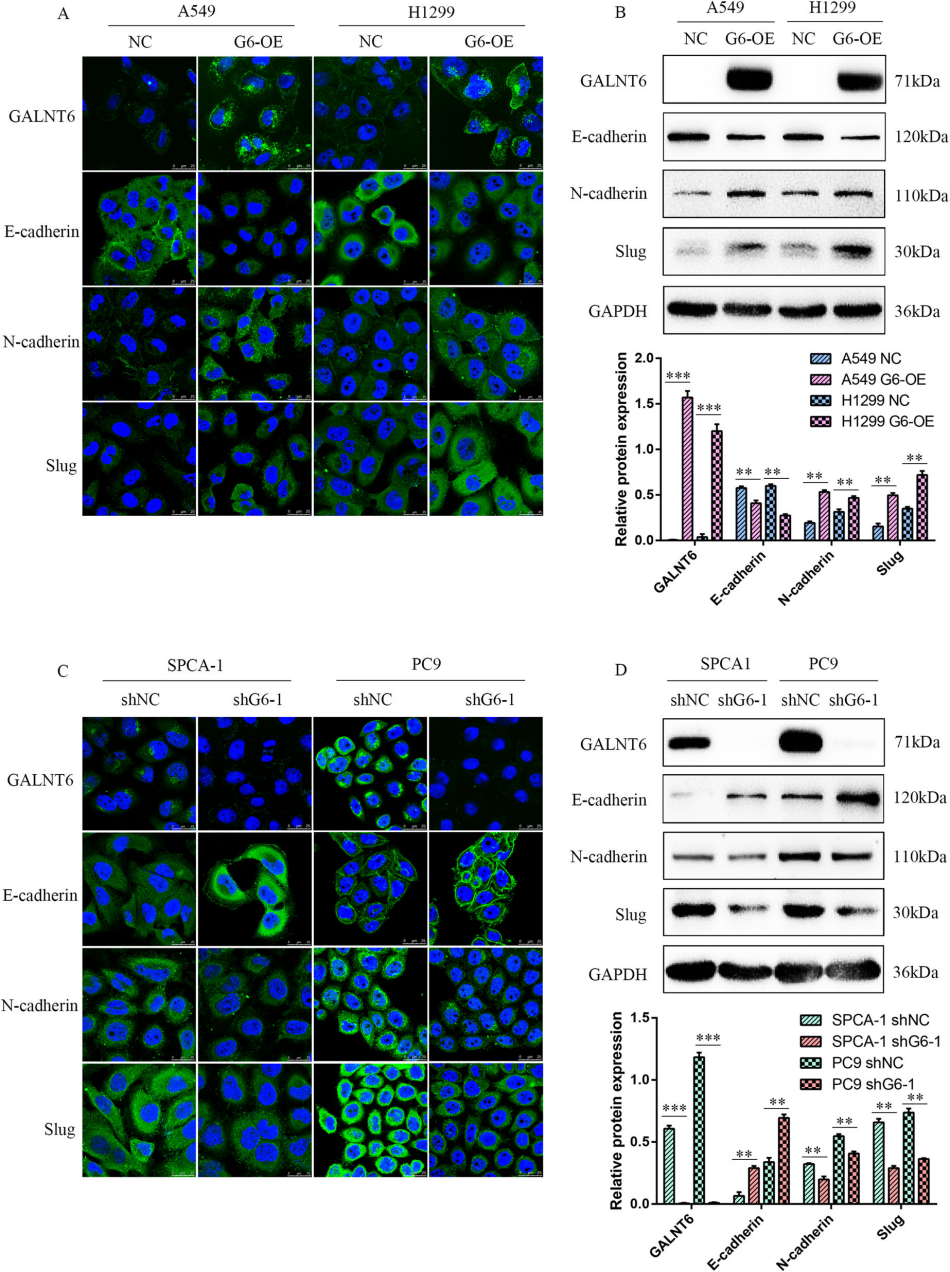
GALNT6 promotes EMT in lung adenocarcinoma cells. A549, H1299, SPCA-1, and PC9 cells were transduced with control lentivirus (NC) or lentivirus for GALNT6 over-expression (G6-OE) or silencing (shNC, shG6-1/2), respectively. GALNT6, E-cadherin, N-cadherin and Slug expression in individual groups of cells were examined by immunofluorescent confocal microscopy and Western blot. **a**, **c** Immunofluorescence analysis. **b**, **d** Western blot analysis. Data are representative images or present as the mean ± SD of each group from three independent experiments. **p < 0.01; ***p < 0.001.

Secondly, we tested the effect of altered GALNT6 expression on EMT in cell lines to assess the ability of cancer cells to metastasize. GALNT6 over-expression decreased E-cadherin expression, but increased N-cadherin and Slug expression in A549 and H1299 cells (*p* < 0.01, Fig. 2a, b). In contrast, GALNT6 silencing upregulated E-cadherin, but down-regulated N-cadherin and Slug expression in SPCA-1 and HC9 cells (*p* < 0.01, Fig. 2c, d). Therefore, GALNT6 may promote EMT in lung adenocarcinoma cells in vitro.

### GALNT6 enhances migration and invasion of lung adenocarcinoma cells

**Fig. 3:**
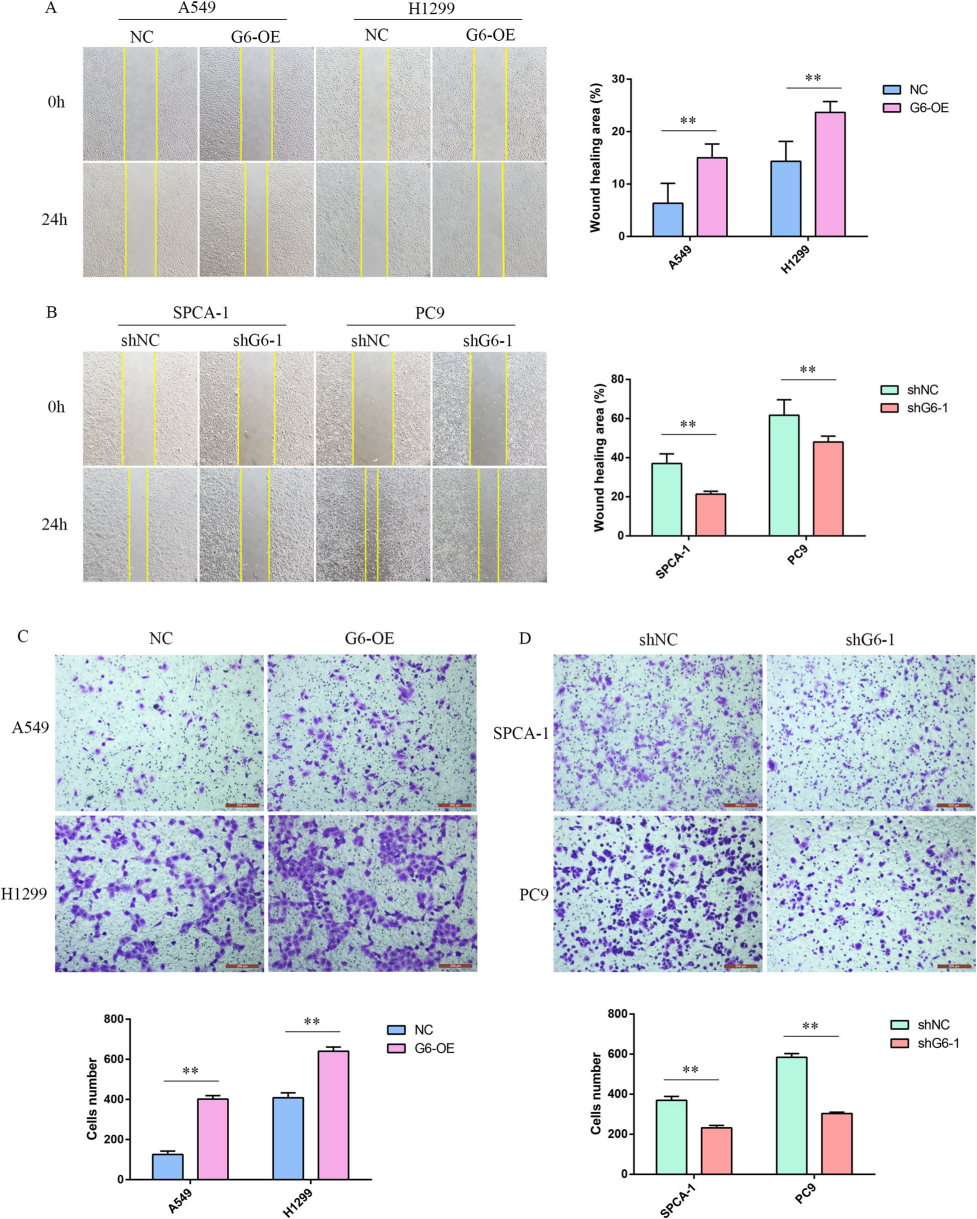
GALNT6 enhances migration and invasion of lung adenocarcinoma cells. **a**, **b** Individual groups of cells were grown up to 80% of confluency and they were wounded. Wound assay is a scratch assay. The wounded areas were photoimaged immediately after wounding and 24 h post wounding. Wound healing area (%) = (0 h Wound area -24h Wound area)/0 h Wound area × 100%. **c**, **d** Transwell invasion analysis of the impact of altered GALNT6 expression on the invasion of lung adenocarcinoma cells. Data are representative images or present as the mean ± SD of each group from three independent experiments. **p < 0.01.

GALNT6 can promote EMT^25,26^, but how it affects tumor invasion and metastasis is not completely known. We then examined the capacity of GALNT6 to change migration and invasion of lung adenocarcinoma cells. GALNT6 over-expression significantly enhanced the wound closure of A549 and H1299 cells while GALNT6 silencing significantly reduced the wound closure of SPCA-1 and PC9 cells (*P* < 0.01, Fig. 3a, b). Similarly, GALNT6 over-expression increased the number of invading A549 and H1299 cells while GALNT6 silencing decreased the number of invading SPCA-1 and PC9 cells (*P* < 0.01, Fig. 3c, d). Thus, GALNT6 enhances the migration and invasion of lung adenocarcinoma cells.

### GALNT6 silencing inhibits lung adenocarcinoma metastasis in vivo

**Fig. 4:**
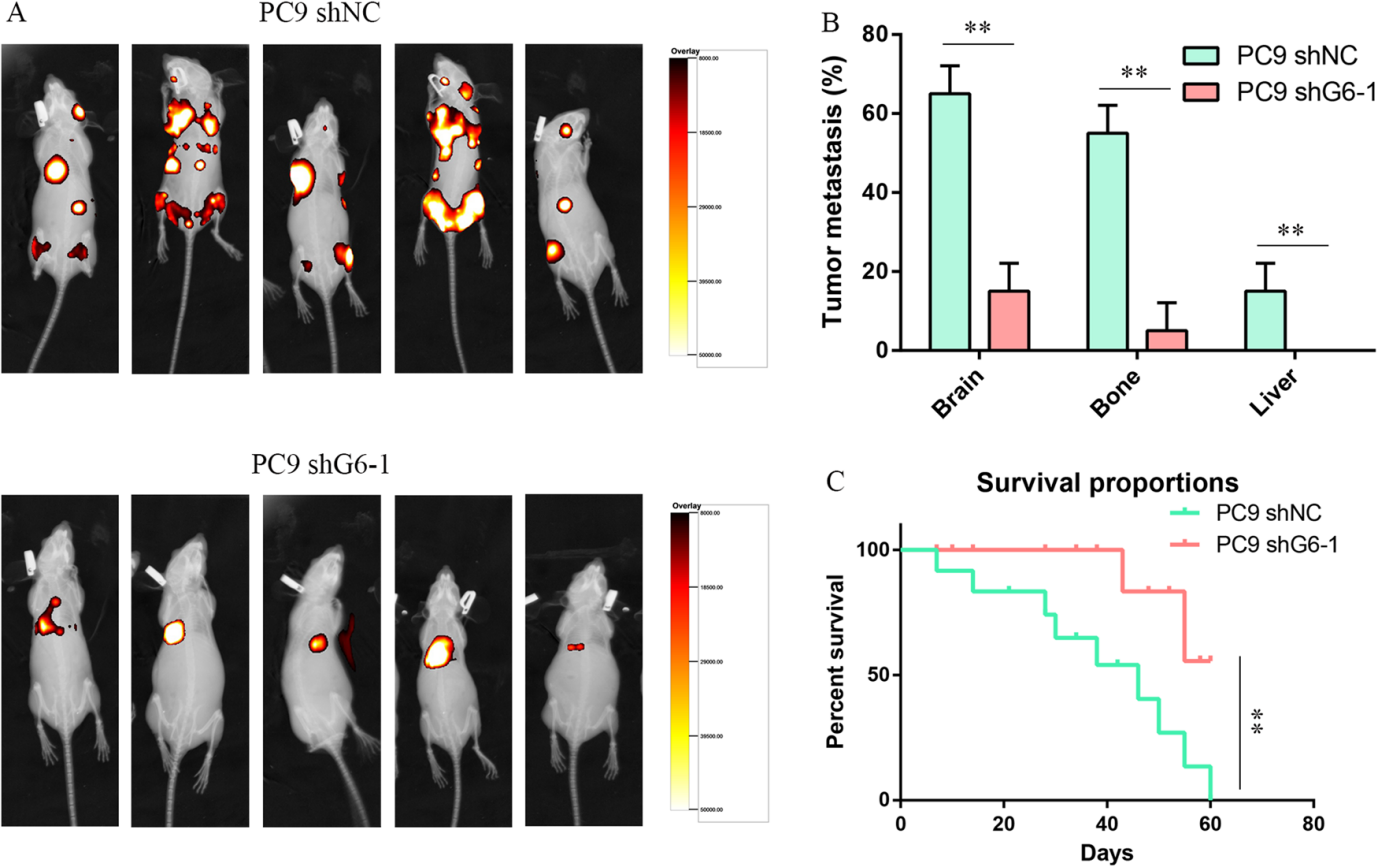
GALNT6 silencing reduces the metastasis of PC9 lung adenocarcinoma cells in mice. PC9 cells were transduced with control lentivirus (shNC) or or silencing (shG6-1), respectively. BALB/c nude mice were injected with PC9-shNC or PC9-shG6-1 cells in their left ventricles. The distribution and sizes of metastatic tumors in the indicated organs were measured by bioluminescent imaging at 30 days post inoculation. The survival of tumor-bearing mice was monitored up to 60 days post inoculation. **a** Bioluminescent images. **b** Quantitative analysis of metastatic tumors in the indicated organs. Tumor metastasis (%) is the percentage of animals with target organ metastasis in the total number of animals. **c** The survival of tumor-bearing mice (*n* = 20 per group). Data are representative images or the mean ± SD of each group (*n* = 20 per group). *p < 0.01.

We further analyzed whether GALNT6 silencing may affect the metastasis of lung adenocarcinoma in vivo. BALB/c nude mice were implanted in their left ventricle with PC9-shNC or PC9-shG6-1 cells transduced with control lentivirus or lentivirus expressing GALNT6-specific shRNA. The distribution and sizes of tumors were monitored by bioluminescence imaging. As shown in Fig. 4a, b, GALNT6 silencing significantly reduced the bioluminescent signals in the brain and bone tissues and abrogated the bioluminescent signals in the liver of mice compared with control PC9-shNC cells (*P* < 0.01). By day 60 post inoculation, while all of the PC9-shNC tumor-bearing mice died, 50% of the mice bearing PC9-shG6-1 tumors survived indicating that GALNT6 silencing prolonged the survival of tumor-bearing mice (*P* < 0.01, Fig. 4c). Therefore, GALNT6 silencing inhibits the metastasis of lung adenocarcinoma in mice.

### GALNT6 O-glycosylates and stabilizes GRP78 in lung adenocarcinoma cells

**Fig. 5:**
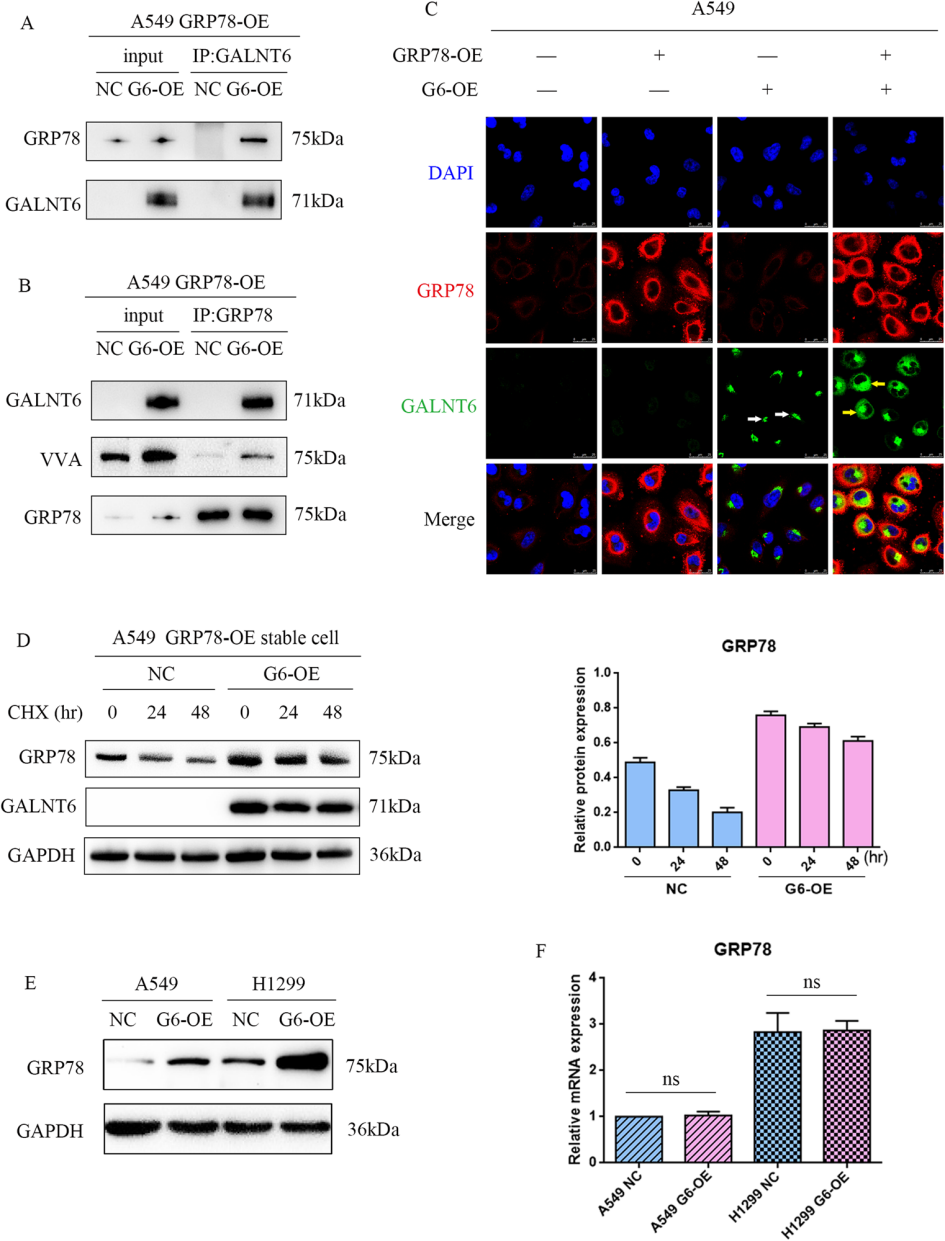
GALNT6 O-glycosylates and stabilizes GRP78 in lung adenocarcinoma cells. A549 cells were transduced with lentivirus for GRP78 over-expression (GRP78-OE), and then transduced with control lentivirus (NC) or GALNT6 over-expression (G6-OE), respectively. The potential direct interaction between GALNT6 and GRP78 was determined by immunoprecipitation using anti-GALNT6 and anti-GRP78. The contained VVA in GRP78 was analyzed by Western blot after using anti-GRP78 precipitation. Furthermore, GRP78 and GALNT6 proteins in individual groups of cells following treatment with cycloheximide (CHX) were tested by Western blot. **a** GALNT6 was pulled down by anti-GALNT6, and then GRP78 and GALNT6 were detected by western blot. **b** GRP78 was pulled down by anti-GRP78, and then GALNT6, VVA and GRP78 were detected by western blot. **c** Immunofluorescent analysis of GALNT6 (green) and GRP78 (red) expression and distribution. We observed that GALNT6 was located in the Golgi if GRP78 was not expressed (white arrows), but in the ER of GRP78 over-expressing cells, indicating that GRP78 over-expression promoted the Golgi-to-ER relocation of GALNT6 (yellow arrows). **d** GALNT6 over-expression stabilized GRP78 protein in A549 cells. **e** GALNT6 over-expression enhanced GRP78 protein expression in A549 and H1299 cells. **f** GALNT6 over-expression did not affect GRP78 mRNA transcription in A549 and H1299 cells. Data are representative images of each group from three independent experiments.

In clinical, cellular and animal studies, GALNT6 has been found to promote the invasion and metastasis of lung adenocarcinoma, but it is unclear how GALNT6 regulates the EMT process to promote tumor metastasis. A recent study showed that GALNT6 O-glycosylates and stabilizes GRP78 in human breast cancer cells, which is associated with apoptotic function of tumors in human^24^. We speculate that GALNT6 may directly interact with GRP78 to regulate the EMT process in lung adenocarcinoma cells. To verify our speculation, we used co-immunoprecipitation and immunofluorescence to study the relationship between GALNT6 and GRP78. We found that anti-GALNT6 antibody precipitated GRP78 (Fig. 5a) and conversely anti-GRP78 also precipitated GALNT6 in A549 stable cells (Fig. 5b). Furthermore, we also observed that GALNT6 was localized preferably in the Golgi (Fig. 5c, white arrows) when GRP78 was absent, but GALNT6 was mainly present in the ER (Fig. 5c, yellow arrows) when GRP78 over-expression was induced, suggesting that GRP78 interacted with GALNT6 to promote its Golgi-to-ER translocation. In addition, the precipitated GRP78 protein contained high levels of biotinylated viciavillosa agglutinin (Fig. 5b), suggesting that GRP78 was highly O-glycosylated in lung adenocarcinoma cells.

Because O-glycosylation is crucial for stabilization of proteins, we examined whether GALNT6 regulates the stabilization of GRP78 protein in A549 cells. After treatment with a protein synthesis inhibitor^24^, cycloheximide (CHX), we found that the relative levels of GRP78 in GALNT6 over-expressing A549 cells were more stable compared with control A549 cells with low GALNT6 expression(Fig. 5d). In fact, induction of GALNT6 over-expression significantly increased GRP78 protein levels (Fig. 5e), but did not significantly alter the levels of GRP78 mRNA transcripts in A549 and H1299 cells (Fig. 5f). These results indicate that GALNT6 O-glycosylated and stabilized GRP78 in lung cancer cells.

### GRP78 promotes EMT, migration, and invasion in lung adenocarcinoma cells

**Fig. 6:**
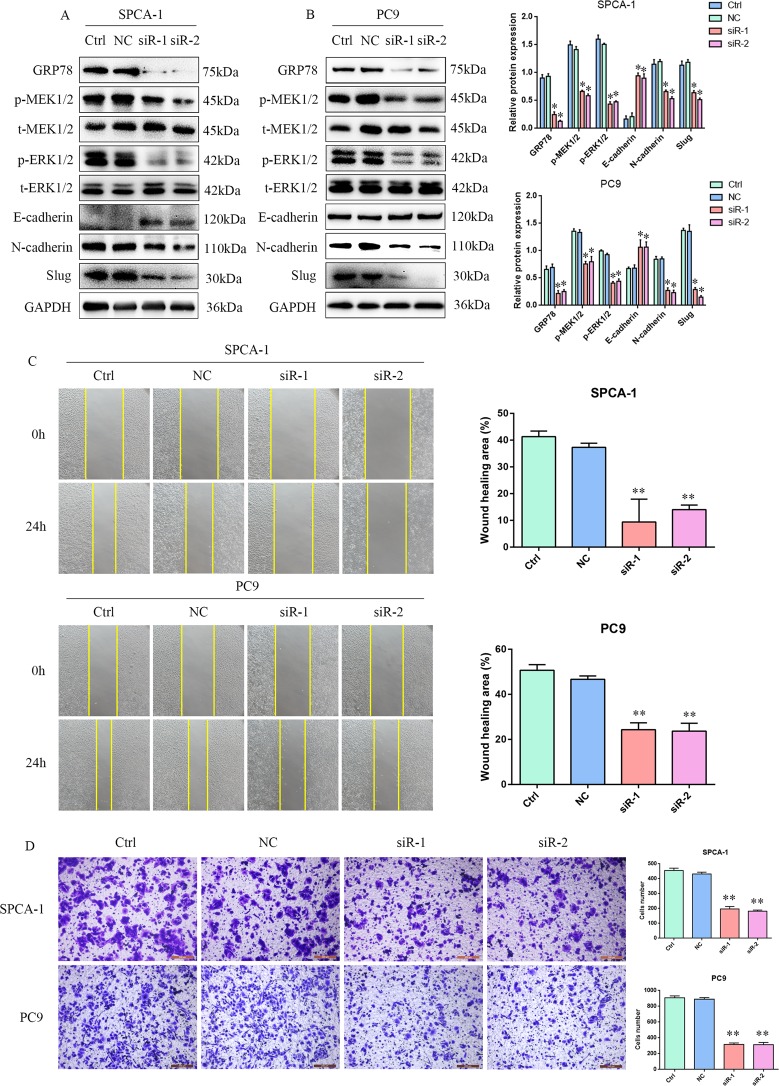
GRP78 promotes EMT, migration and invasion in lung adenocarcinoma cells. SPCA-1 and PC9 cells were transfected with control siRNA (NC) or GRP78-specific siRNAs (SiR-1/2). The relative levels of GRP78, MEK1/2 and ERK1/2 expression and MEK1/2 and ERK1/2 phosphorylation as well as E-cadherin, N-cadherin and Slug expression were determined by Western blot. The migration and invasion capabilities were determined by wound closure and transwell invasion assays. **a**, **b** GRP78 silencing inhibited the EMT process and attenuated the MEK/ERK signaling. **c**, **d** GRP78 silencing inhibited the migration and invasion lung adenocarcinoma cells. Wound healing area (%) = (0 h Wound area -24h Wound area)/0 h Wound area×100%. Data are representative images or present as the mean ± SD of each group of cells from three separate experiments. *p < 0.05; **p < 0.01.

To assess the regulation of GRP78 on EMT in lung adenocarcinoma cells, SPCA-1 and PC9 cells were transfected with GRP78-specific or control siRNA to examine its impacts on the EMT process, migration and invasion. As shown in Fig. 6, GRP78 silencing reduced the expression of N-cadherin and Slug, but increased the expression of E-cadherin in SPCA-1 and PC9 cells (*P* < 0.05, Fig. 6a, b). Furthermore, GRP78 silencing significantly reduced the wound healing and the number of invaded SPCA-1 and PC9 cells (*P* < 0.01, Fig. 6c, d). Such data suggest that GRP78 may enhance the EMT, migration and invasion of lung adenocarcinoma cells.

### GRP78 promotes EMT by enhancing the MEK/ERK signaling

**Fig. 7:**
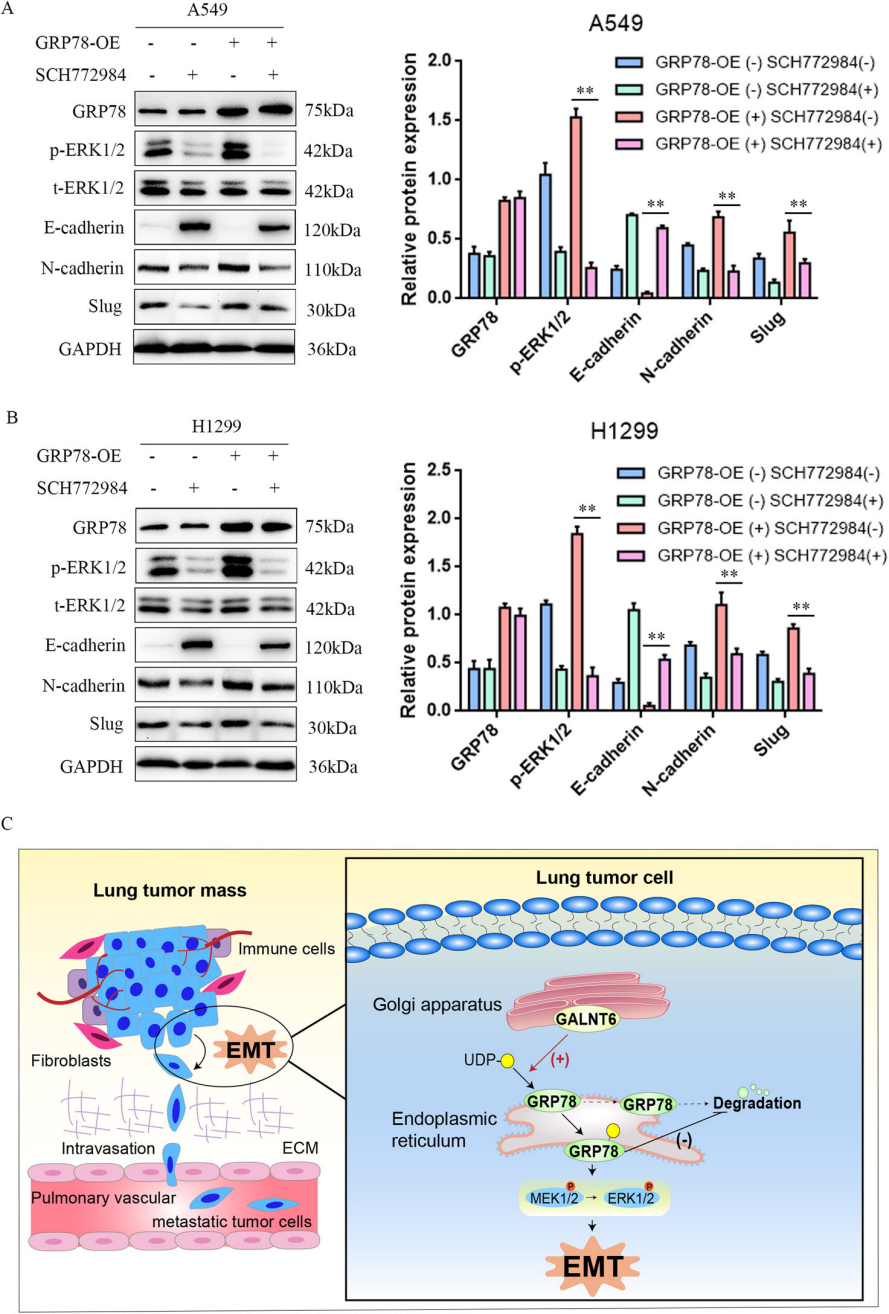
GRP78 promotes EMT by enhancing MEK/ERK signaling pathway. A549 and H1299 cells were transduced with control lentivirus or lentivirus for GRP78 over-expression (GRP78-OE). The cells were treated with, or without, SCH772984 and the relative levels of GRP78, ERK1/2, E-cadherin, N-cadherin and Slug expression and ERK1/2 phosphorylation were determined by Western blot. **a**, **b** GRP78 over-expression promoted the EMT process in lung adenocarcinoma cells by activating the ERK signaling, while treatment with SCH771984 not only inhibited the ERK1/2 phosphorylation, but also attenuated the EMT process regardless of GRP78 over-expression. **c** A diagram illustrates the potential mechanisms by which GALNT6 promotes the metastasis of lung adenocarcinoma. GALNT6 O-glycosylates and stabilizes GRP78 to activate the MEK/ERK signaling, and subsequently leads to the EMT process in lung adenocarcinoma cells. Data are representative images or present as the mean ± SD of each group from three independent experiments. **p < 0.01.

As shown in Fig. 6, we observed that GRP78 silencing decreased MEK1/2 and ERK1/2 phosphorylation (Fig. 6a, b). Following induction of stable GRP78 over-expression in A549 and H1299 cells (Supplementary Fig. 2), we observed that GRP78 over-expression increased ERK1/2 phosphorylation and EMT process (Fig. 7a, b), which were dramatically mitigated and abrogated by treatment with SCH772984, an ERK inhibitor in both A549 and H1299 cells regardless of GRP78 over-expression (Fig. 7a, b). Therefore, GRP78 promotes the EMT process by enhancing the MEK1/2/ERK1/2 signaling in lung adenocarcinoma cells.

To further investigate our findings, we conducted simple experiments in vitro and in vivo. When stable GALNT6 over-expression A549 cells treated with GRP78-siRNA, we observed that ERK1/2 phosphorylation and the EMT process were dramatically mitigated and abrogated regardless of GALNT6 over-expression (Supplementary Fig. 3). Concurrent with our in vitro data, immunohistochemistry staining of xenograft tissue slides indicated GRP78 and p-ERK1/2 downregulation upon GALNT6 knockdown (Supplementary Fig. 4). We also observed that GALNT6, GRP78, and p-ERK1/2 were upregulated in lung cancer tissues compared with adjacent normal lung tissues (Supplementary Fig. 5). Altogether, these results suggest that elevated GALNT6-GRP78-MEK1/2/ERK1/2 signaling (Fig. 7c) may essentially contribute to lung cancer metastasis.

## Discussion

Adenocarcinoma is the most common lung cancer in women and in non-smokers^4,5,27^. Because of its high morbidity and mortality, many studies attempt to discover new ways for early diagnosis and to identify novel therapeutic targets.

In the current study, we found that GALNT6 expression was upregulated in human lung adenocarcinoma tissues, which was significantly correlated with higher tumor stage, lymph node metastasis, TNM stage and poor prognosis. Furthermore, higher levels of GALNT6 expression were associated with poor overall survival of 720 patients with lung adenocarcinoma in database. Such findings extended previous observations in breast and gastric cancers^12,28^ and suggest that higher levels of GALNT6 expression may predict the outcome of lung adenocarcinoma in the clinic.

Cancer recurrence and distant metastasis are attributed to increased EMT process in cancer cells^15^. The EMT program was originally described as an integral part of morphogenesis in embryonic development, and later was observed in several pathogenesis events, including wound healing, fibrosis, and cancer metastasis^29^. But the role of EMT in tumor metastasis has been debated for nearly two decades^30,31^. Various studies in cultured human cancer cell lines, mouse tumor models, and human tumor samples have demonstrated that induction of an EMT program allows carcinoma cells to lose cell-cell junctions, degrade local basement membrane via elevated expression of various matrix-degrading enzymes, and thus support their migration and invasion as single cells^32,33^. A few studies failed to detect EMT in disseminating tumor cells using certain EMT reporters, and it therefore raises the possibility that certain carcinoma cells could disseminate and form distant metastases without undergoing EMT^34^. Given the difficulty to sensitively detect activation of the transient and partial EMT program in vivo, further studies are needed to develop more efficient EMT reporters to further evaluate the involvement of EMT in metastasis to clearly settle this decade-old debate.

Emerging data indicate that GALNT6 promotes EMT in human breast and prostate cancers^25,26^. In this study, we found that GALNT6 enhanced EMT in lung adenocarcinoma cells. Mechanistically, GALNT6 over-expression in lung adenocarcinoma cells increased N-cadherin and Slug expression, but decreased E-cadherin expression while GALNT6 silencing had opposite effects. Consequently, GALNT6 promoted the in vitro wound closure and invasion as well as metastasis of lung adenocarcinoma in mice. These results suggest that high levels of GALNT6 may promote EMT in epithelial cancer cells to acquire migration and invasion capacity for metastasis in distant organs. Since distant metastasis is the key for lung cancer-related death, targeting GALNT6 may inhibit the metastasis of lung adenocarcinoma.

GALNT6 initiates mucin-type O-glycosylation and is critical for post-translational processing of glycoproteins and their stability^35^. A previous study reports that GALNT6 O-glycosylates and stabilizes Mucin 1 (MUC1), regulating the EMT process in breast cancer cells^25^. In addition, GALNT6 enhances transformational potentials and invasiveness of breast cancer cells by O-glycosylation of fibronectin^36^. Furthermore, GALNT6 is essential in O-glycosylation and stabilization of Mucin 4 protein, and critical for the EMT process in pancreatic cancer^37^. Not all substrates of GALNT6 have been defined, a recent report indicates that GRP78 is one of the substrates in breast cancer cells^24^, but it is unclear whether GALNT6 can O-glycosylate GRP78 to regulate the EMT process in lung adenocarcinoma cells. In this study, we found that GALNT6 O-glycosylated and stabilized GRP78, and enhanced the activation of MEK/ERK signaling in lung adenocarcinoma cells. GALNT6 directly interacted with GRP78 and its over-expression upregulated GRP78 and enhanced MEK1/2 and ERK1/2 phosphorylation and EMT in lung adenocarcinoma cells. These data are in agreement with previous reports^38–40^ and indicate that GRP78 positively regulates the invasion and metastasis of malignant tumors. It is possible that the upregulated GRP78 may promote MMP-2, MMP-9 and uPA expression to increase ECM degradation, leading to cancer cell invasion^38^. Alternatively, GRP78 over-expression may enhance TGF-β/Smad2/3 signaling to promote EMT^33^.These, together O-glycosylation and stabilization of fibronectin and other substrates, may contribute to the effect of GALNT6 and GRP78 on EMT and metastasis of lung adenocarcinoma^25,26,35–40^. Therefore, GALNT6-GRP78-MEK1/2/ERK1/2 signaling regulates EMT and metastasis of lung adenocarcinoma.

However, our current study does have some limitations. Firstly, the clinical sample size was relatively smaller, particularly for the lack of sufficient numbers of samples with distant metastasis. Secondly, the effects of GALNT6 silencing appeared to be greater than those of GRP78 silencing, which may stem from GALNT6 O-glycosylating other substrates. Conceivably, further identification of new substrates of GALNT6 is necessary to clarify the roles of GALNT6 in metastasis of lung adenocarcinoma.

## Supplementary information

Supplementary Information

supplement figure 1

supplement figure 2

supplement figure 3

supplement figure 4

supplement figure 5
