# Supplementary material for: GALNT6 promotes invasion and metastasis of human lung adenocarcinoma cells through O-glycosylating chaperone protein GRP78
Source: Cell Death Dis. 2020 May 11;11(5):352. doi: 10.1038/s41419-020-2537-6 (PMC7214460; doi:10.1038/s41419-020-2537-6)
Supplement: Supplementary file 1 — Supplementary Information [file 41419_2020_2537_MOESM1_ESM.docx]

Supplementary Information

Supplementary Figure Legends:

Supplementary Figure 1. GALNT6 protein expression in different lung adenocarcinoma cells.

A549, H1299, SPCA-1 and PC9 cells were transduced with control lentivirus (NC) or lentivirus for GALNT6 over-expression (G6-OE) or silencing (shNC, shG6-1/2), respectively. (A)Western blot analysis of GALNT6 expression in the indicated cells. (B) Western blot analysis of GALNT6 overexpressing or silenced in tumor cells. Data are representative images of each group of cells from three separate experiments.

Supplementary Figure 2. Western blot analysis of GRP78 over-expressing in tumor cells. A549 and H1299 cells were transduced with control lentivirus (NC) or lentivirus for GRP78 over-expression (GRP78-OE). Data are representative images of each group of cells from three separate experiments.

Supplementary Figure 3. GALNT6 promotes EMT by enhancing GRP78/ ERK1/2 signaling pathway. A549 cells were transduced with lentivirus for GALNT6 over-expression (GALNT6-OE). The cells were treated with or without GRP78-siRNA and the relative levels of ERK1/2 phosphorylation, ERK1/2, E-cadherin, N-cadherin and Slug expression were detected by Western blot. GALNT6 over-expression promoted the EMT process in lung adenocarcinoma cells by activating the ERK signaling, while treatment with GRP78-siRNA not only inhibited the ERK1/2 phosphorylation, but also attenuated the EMT process regardless of GALNT6 over-expression.

Supplementary Figure 4. GALNT6 knockdown decreases GRP78/ERK1/2 signaling in vivo. Representative images of immunohistochemical (IHC) staining for GALNT6, GRP78, and p-ERK1/2 in PC9-shNS/shG6-1 xenograft tumor tissues.

Supplementary Figure 5. GALNT6/GRP78/ERK1/2 signaling was elevated in lung cancer tissues compared with adjacent normal lung tissues. Representative images of immunohistochemical (IHC) staining for GALNT6, GRP78, and p-ERK1/2 in lung adenocarcinoma tissue (T) and adjacent normal lung tissues (N) from lung cancer patients.
