# Supplementary figures and images for: GALNT6 promotes invasion and metastasis of human lung adenocarcinoma cells through O-glycosylating chaperone protein GRP78
Source: Cell Death Dis. 2020 May 11;11(5):352. doi: 10.1038/s41419-020-2537-6 (PMC7214460; doi:10.1038/s41419-020-2537-6)

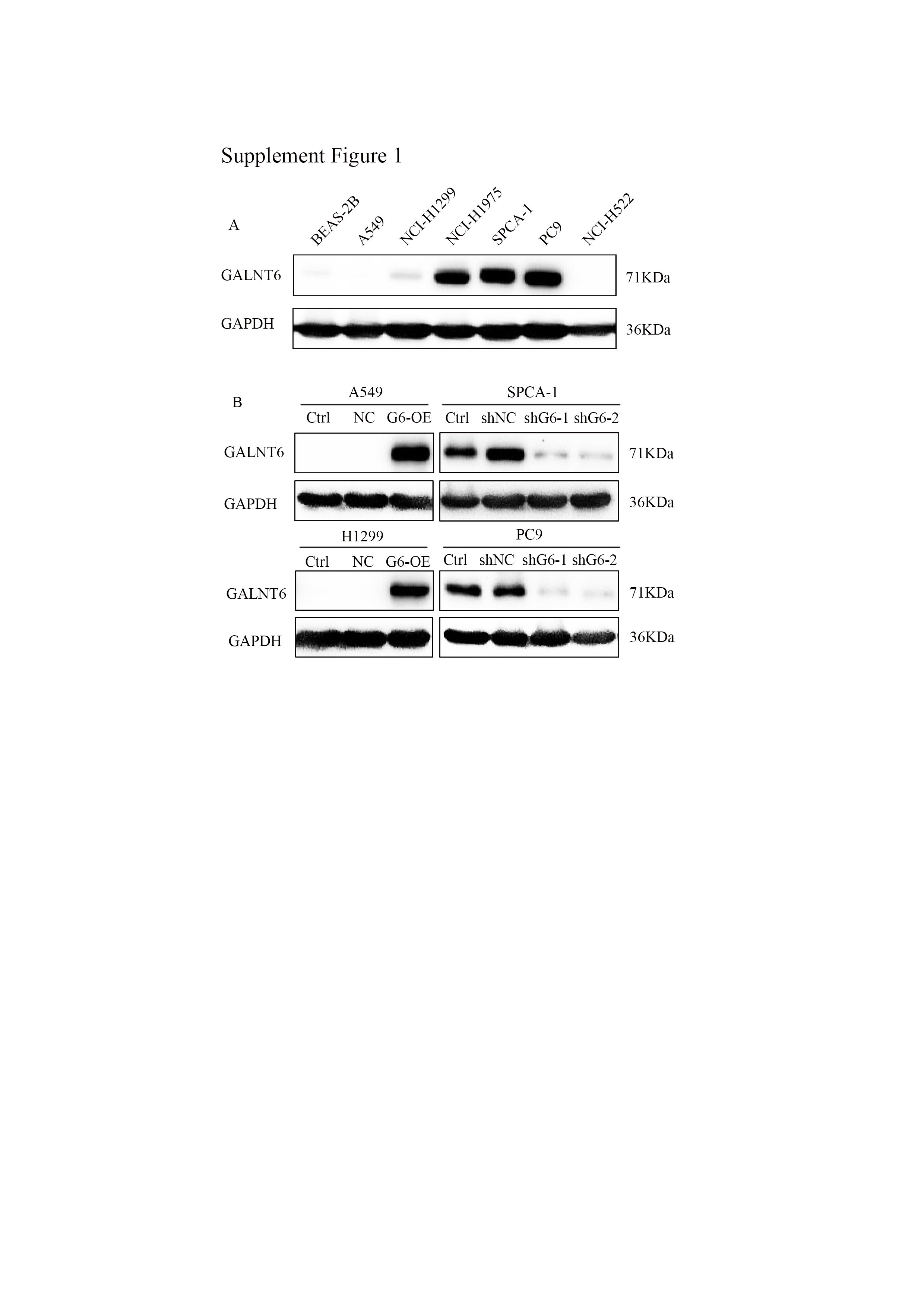

Supplement: Supplementary file 2 — supplement figure 1 [file 41419_2020_2537_MOESM2_ESM.tif]

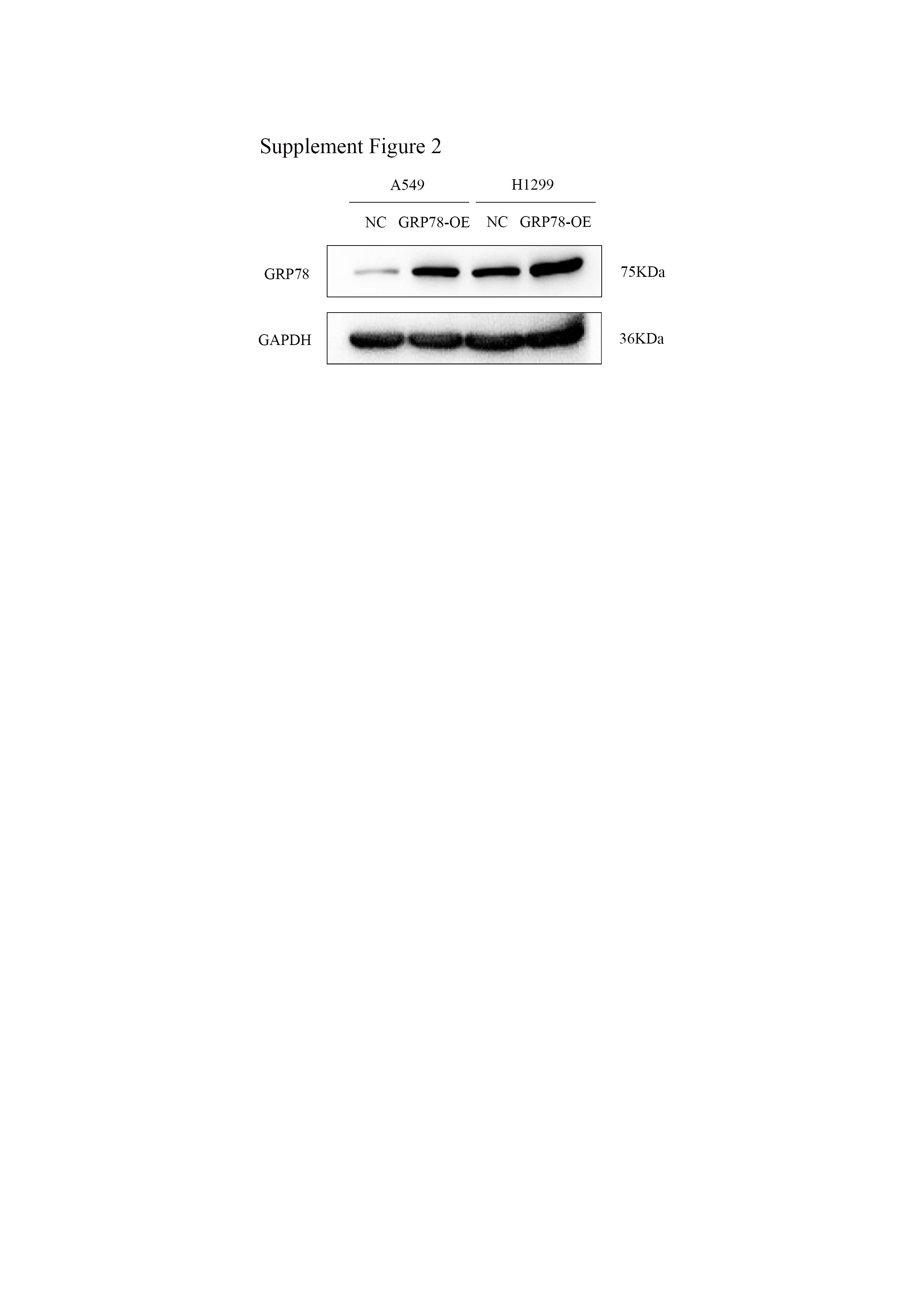

Supplement: Supplementary file 3 — supplement figure 2 [file 41419_2020_2537_MOESM3_ESM.tif]

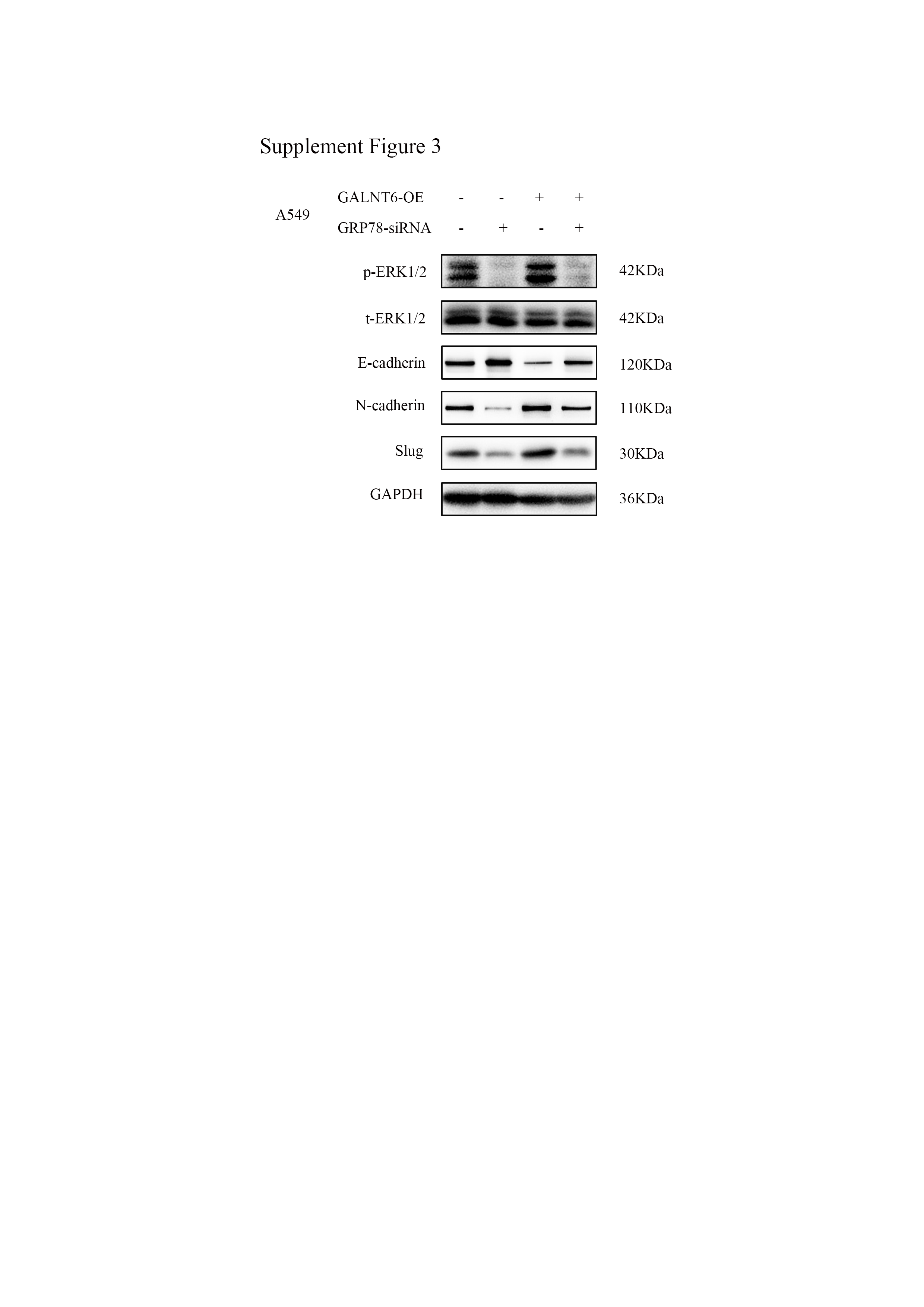

Supplement: Supplementary file 4 — supplement figure 3 [file 41419_2020_2537_MOESM4_ESM.tif]

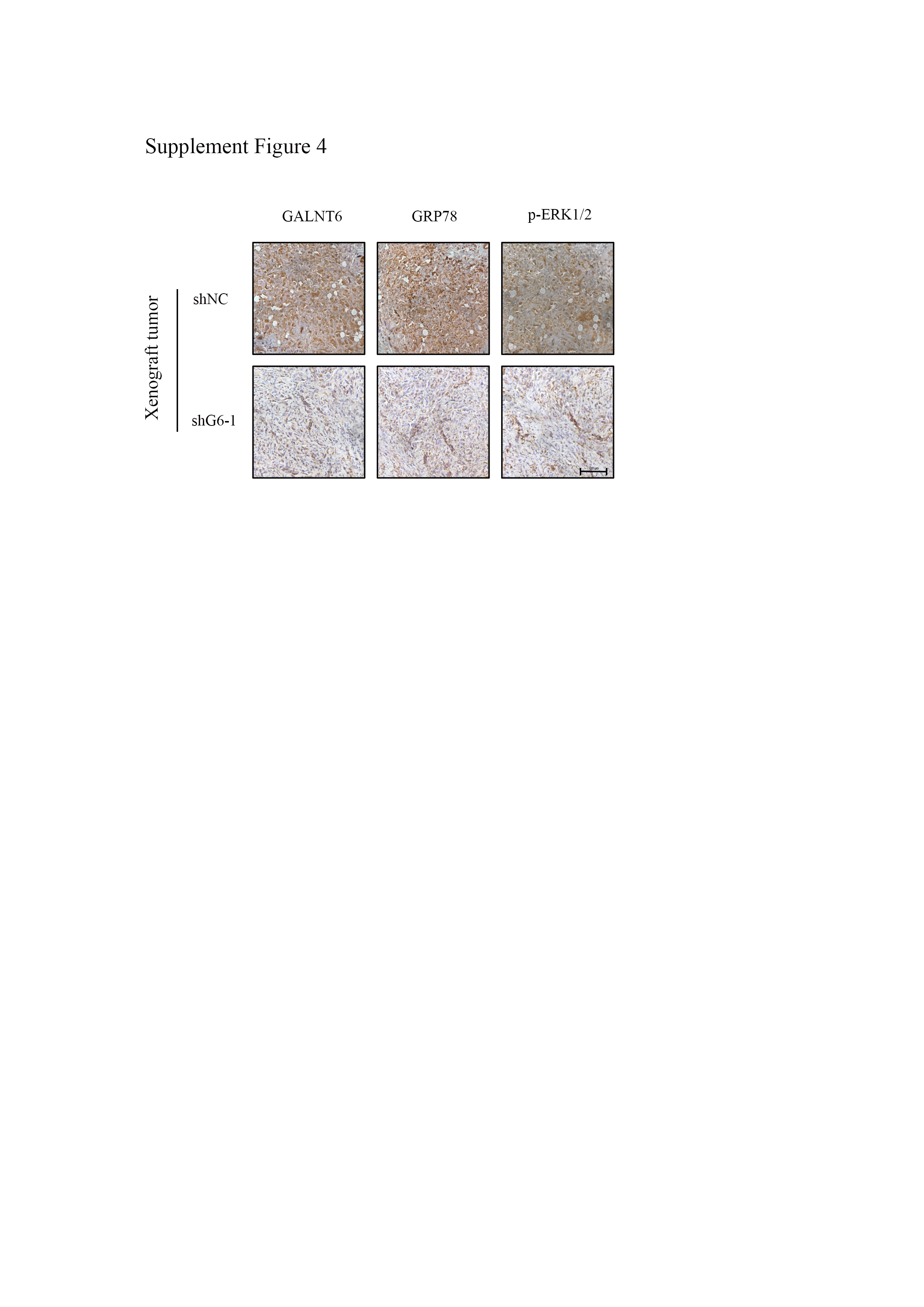

Supplement: Supplementary file 5 — supplement figure 4 [file 41419_2020_2537_MOESM5_ESM.tif]

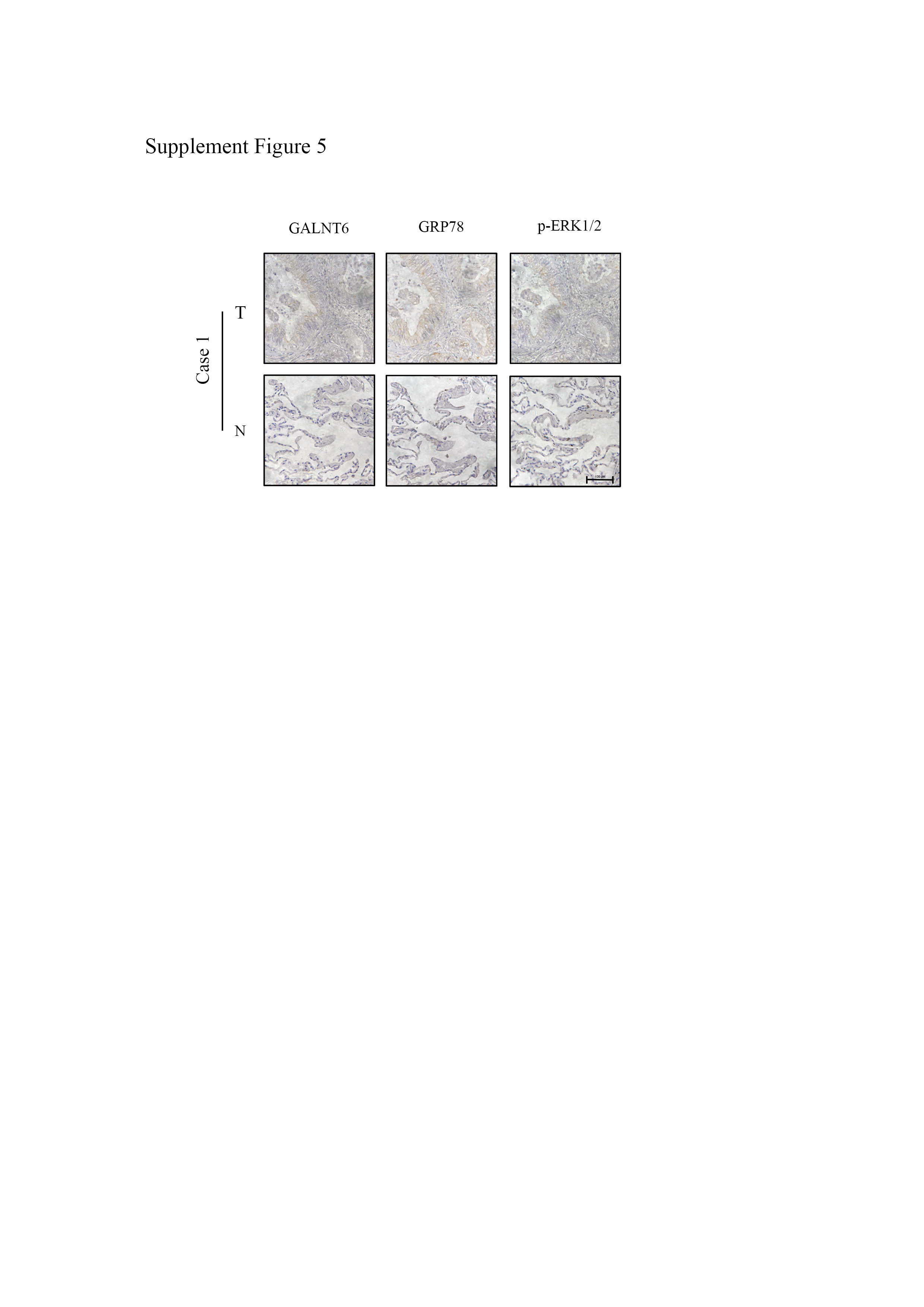

Supplement: Supplementary file 6 — supplement figure 5 [file 41419_2020_2537_MOESM6_ESM.tif]
